# Effect of inter-train interval on the induction of repetition suppression of motor-evoked potentials using transcranial magnetic stimulation

**DOI:** 10.1371/journal.pone.0181663

**Published:** 2017-07-19

**Authors:** Minna Pitkänen, Elisa Kallioniemi, Petro Julkunen

**Affiliations:** 1 Department of Clinical Neurophysiology, Kuopio University Hospital, Kuopio, Finland; 2 Department of Neuroscience and Biomedical Engineering, Aalto University School of Science, Espoo, Finland; 3 Department of Clinical Radiology, Kuopio University Hospital, Kuopio, Finland; 4 Department of Applied Physics, University of Eastern Finland, Kuopio, Finland; University of Toronto, CANADA

## Abstract

Repetition suppression (RS) is evident as a weakened response to repeated stimuli after the initial response. RS has been demonstrated in motor-evoked potentials (MEPs) induced with transcranial magnetic stimulation (TMS). Here, we investigated the effect of inter-train interval (ITI) on the induction of RS of MEPs with the attempt to optimize the investigative protocols. Trains of TMS pulses, targeted to the primary motor cortex by neuronavigation, were applied at a stimulation intensity of 120% of the resting motor threshold. The stimulus trains included either four or twenty pulses with an inter-stimulus interval (ISI) of 1 s. The ITI was here defined as the interval between the last pulse in a train and the first pulse in the next train; the ITIs used here were 1, 3, 4, 6, 7, 12, and 17 s. RS was observed with all ITIs except with the ITI of 1 s, in which the ITI was equal to ISI. RS was more pronounced with longer ITIs. Shorter ITIs may not allow sufficient time for a return to baseline. RS may reflect a startle-like response to the first pulse of a train followed by habituation. Longer ITIs may allow more recovery time and in turn demonstrate greater RS. Our results indicate that RS can be studied with confidence at relatively short ITIs of 6 s and above.

## Introduction

Transcranial magnetic stimulation (TMS) is a non-invasive method which externally activates the cortex of the brain [1]. TMS may be applied to evaluate the excitability of the motor system due to its ability to induce measurable motor-evoked potentials (MEPs), i.e., muscle twitches, when the stimulation is targeted to the excitable motor areas of the cortex with a sufficient stimulation intensity (SI) [2,3]. TMS can be applied as single pulses, paired pulses, or trains of pulses. Previous stimulation pulses can transiently influence the MEP amplitudes if the inter-stimulus interval (ISI) is short. On the other hand, long trains of repetitive TMS can induce long-term effects on the cortex [4]. Previous single-pulse TMS studies on potential carry-over effects on MEP amplitudes have reported that these have no major impact at ISIs of at least 5 s [5]. In addition to ISI, inter-train interval (ITI) may affect the cortical excitability or inhibition or both. For example, ITI has been shown to influence the plastic effects of high-frequency repetitive TMS [6].

When single-pulses were delivered in short trains at an ISI of 1 s, the second, third, and fourth MEPs were found to suppress in amplitude in comparison to the first MEP in the train [7]. This phenomenon, repetition suppression (RS), or the habituation of a startle response, is observed as a rapid decrement of response amplitudes which may show a trend towards gradual recovery [7]. RS is thought to represent a general mechanism for the brain’s reaction to external repeated stimuli. RS has been well characterized in the auditory-evoked potentials (AEPs) induced by auditory stimuli [8–10] and more recently in the motor responses induced by TMS [7,11,12]. The MEP amplitudes after second, third, and fourth stimuli have been reported to decline to 40–58% of the first MEP amplitude [7,12]. RS has also been demonstrated in the cortical electroencephalography N100 responses generated by TMS [7] and by sensory stimuli [13,14]. RS in the AEPs has been observed to last at least until the 30th stimulus [8]. However, any direct comparison between the auditory, sensory, and motor systems is complicated by the potentially different synaptic organizations of these systems. On the other hand, the auditory sensory and the primary motor networks likely are linked since they communicate with each other [11].

The physiological origin of RS in the motor system is not fully understood, although previously a peripheral source was ruled out by electric stimulation of the medianus nerve [7]. The high-amplitude of the first evoked potential may be due to a startle or an arousal effect, where the brain reacts intensely to a new and surprising stimulus. After arousal, the brain may interpret the subsequent repeated stimuli as less important and suppress reactivity to prevent an overreaction [15]. Several mechanisms have been postulated [16,17]. RS might be modulated by the inhibitory system, because of the lengthening of TMS-evoked corticospinal silent periods during RS, suggesting the possibility of continuously increasing GABAergic inhibition during stimulation trains [12]. In addition, RS has been observed in the mental imagery of movement [18] and in the repetition of hand gestures [19]. For these reasons, RS has been claimed to represent a general short-term mechanism in the cortex to external stimuli such as TMS [7].

Indications of abnormal sensory RS effect have been found in some diseases, e.g., in schizophrenia [20] and in migraine [14] and it might also be involved in some symptoms of age-associated memory impairment [21]. Similar findings could potentially be found in the motor system RS and thus, it could provide a novel, non-invasive biomarker for diseases affecting the cortical motor control, e.g., myoclonus epilepsies or Parkinson’s disease.

Thus far, in RS-MEP studies, the number of the stimuli in the trains has been limited to four and the ITI has been 17 s in order to demonstrate the appearance of the phenomenon. Such protocol has been derived from RS studies on the auditory system [7]. However, the influence of the number of pulses and the duration of the ITI has not been investigated. If the ITI does not affect RS, the measurement time in RS-MEP studies can be reduced. Therefore, in the present study, we studied the effect of ITI on the appearance and effect size of RS in healthy volunteers with neuronavigated TMS (nTMS). We hypothesized that the break between trains of stimuli would be crucial for the appearance of RS, with the effects of RS being emphasized at the longest ITIs.

## Materials and methods

Fifteen healthy right-handed volunteers (8 males, age: 24–62 years) participated in the study. Written informed consent was received from each of them and none had contraindications for nTMS or magnetic resonance imaging (MRI). The study was approved by the research ethics committee of the Kuopio University Hospital (78/2014).

Structural T1-weighted MRIs were collected with a 3T MRI scanner (Philips Achieva 3.0T TX, Philips, Eindhoven, The Netherlands) and utilized in the neuronavigation of TMS. A biphasic pulse wave-form with an air-cooled figure-of-eight coil was used in the stimulation (NBS 4.3.1, Nexstim Plc., Helsinki, Finland). An nTMS-compatible electromyography device recorded the muscle activity from the first dorsal interosseous (FDI) muscle of the dominant, right hand at rest. During the experiments, subjects were seated comfortably, wore earplugs, and watched a silent video on the screen in front of them.

**Fig 1:**
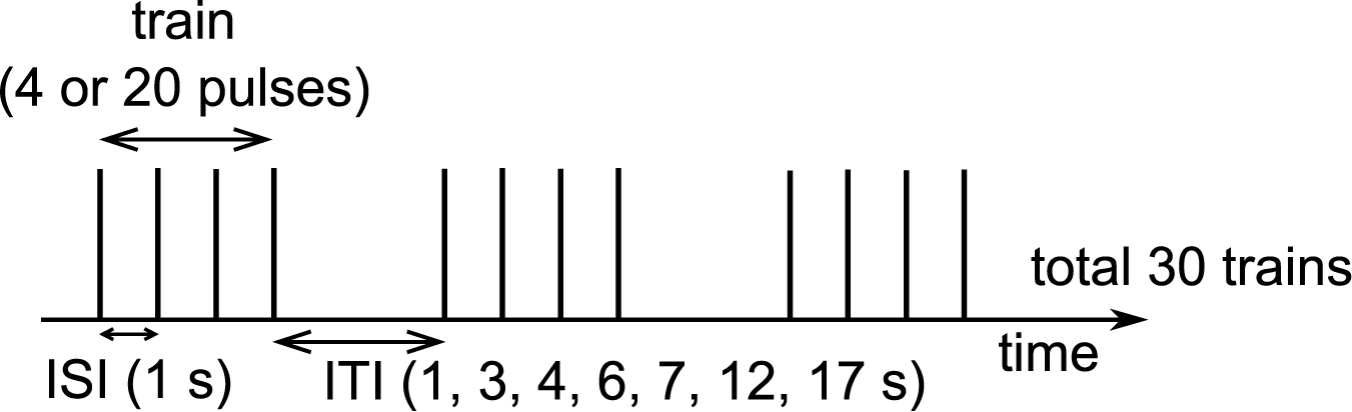
The stimulation protocol included eight sequences. In the first seven sequences, 30 trains consisted of four or twenty pulses. The inter-train interval (ITI) was determined as an interval between the last pulse in a train and the first pulse in the next train. The ITIs were 3, 4, 6, 7, 12, and 17 s and an inter-stimulus interval (ISI) was 1 s. In the last sequence, 120 pulses were applied with an ISI of 1 s. When the last sequence was divided into 4-pulse trains, the ITI was equal to ISI and the number of trains was 30. The sequences were applied in a random order except the 120 pulses/train sequence which was always given last.

The stimulation began by searching for the FDI hotspot, i.e., the cortical location on the left hemisphere capable of inducing the maximum MEP for the FDI muscle. The optimal direction was determined by rotating the coil at the hotspot and choosing the direction that induced the largest MEP amplitudes. Thereafter, the resting motor threshold (rMT) was measured at the hotspot using the iterative threshold assessment tool integrated into the stimulation software. The tool resembles the threshold hunting algorithm [22,23]. The subsequent stimulation pulses were targeted to the hotspot at an intensity of 120%-rMT. The stimulation protocol (Fig 1) consisted of the following sequences applied in a randomized order: 30 trains of four pulses given at ITIs of 3, 4, 6, 7, 12, and 17 s, and 30 trains of 20 pulses given at ITI 17 s. There were short breaks of a few minutes between the sequences. The ITI was determined from the last pulse in a train to the first pulse of the subsequent train (Fig 1). The ISI was 1 s within all the trains. In addition, one train of 120 pulses at ISI of 1 s was applied at the end of each experiment. The sequence was placed at the end of the session due to the possibility of induction of inhibitory repetitive TMS effects [24]. This sequence was analyzed by two different techniques: it was either i) divided into 4-pulse trains as was done with the other sequences to resemble the use of 1 s ITI (hereafter referred to as a sequence with ITI of 1 s) or ii) it was treated as a long train of 120 pulses. This sequence had to be excluded from the analysis of one subject due to the low number of induced MEPs. In addition, the sequence with 12 s ITI was not measured in one subject due to technical reasons.

The preprocessing of the MEP data was performed using MegaWin software (version 3.1, Mega Electronics Ltd, Kuopio, Finland). MEPs lower than 50 μV in amplitude were considered as no responses and only the MEPs occurring in a resting muscle were accepted in the analyses. The muscle activity preceding stimuli was inspected visually. Approximately 2% of the MEPs were excluded from the analysis. All the MEP amplitudes within trains were first averaged over all trains based on their ordinal position within a train. Then the averaged MEPs were normalized to the first one in the train. Therefore, a low normalized MEP amplitude is indicative of a high RS effect, whereas a normalized MEP amplitude close to 1 represents a lack of any major RS effect. The Linear Mixed Model with Bonferroni post-hoc analysis was used to assess the effects of the stimulus number within a train and ITI separately for the 4-pulses/train, 20-pulses/train, and 120-pulses/train sequences. In the 120-pulse train, normalized single-trial MEPs were used in the comparisons. A *p*-value of less than 0.05 indicated statistical significance. In the statistical analyses, SPSS Statistics 23 (IBM Corporation, Somers, NY, USA) was utilized.

## Results

The individual rMTs were 40±9% of the maximum stimulator output.

**Fig 2:**
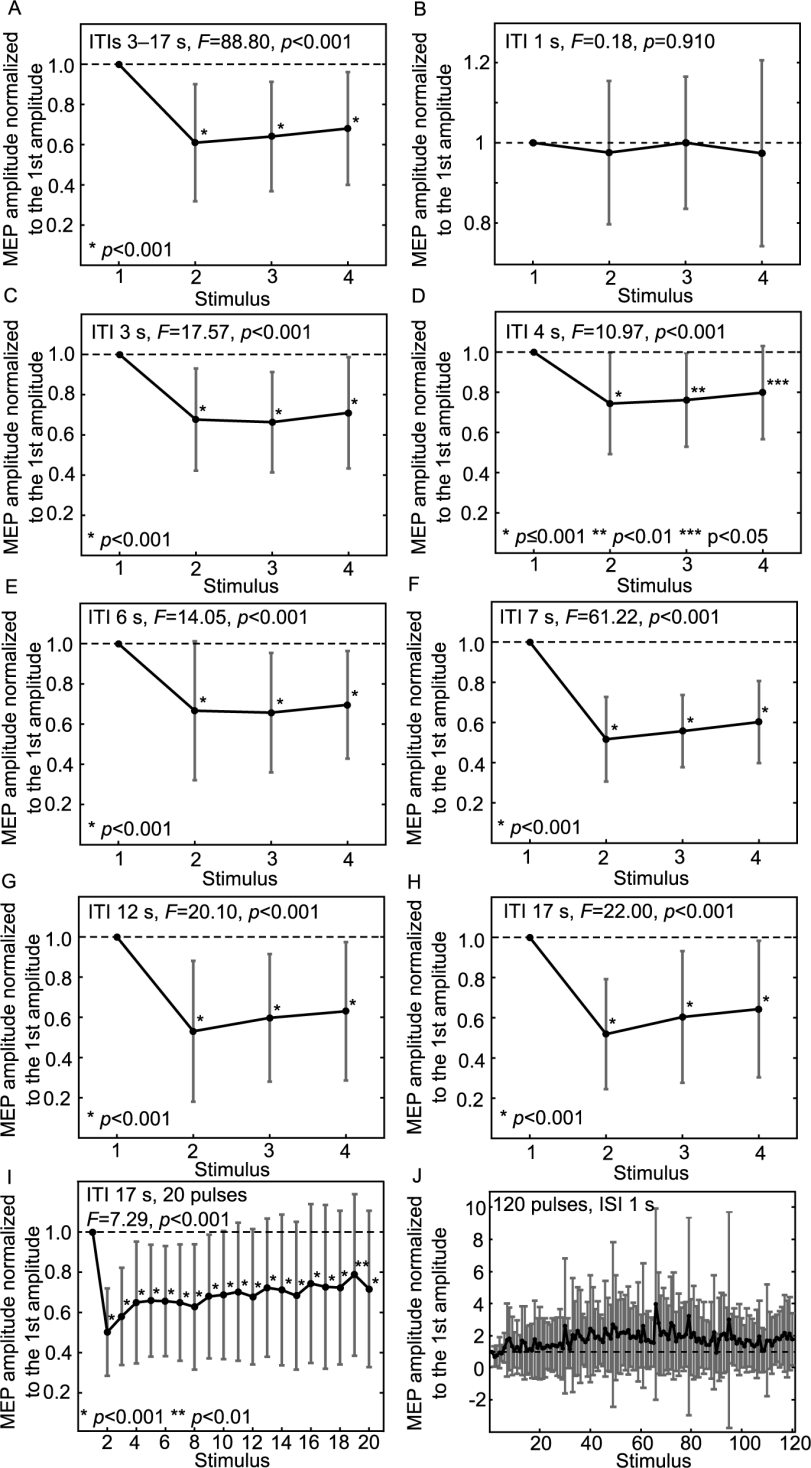
Repetition suppression of the motor-evoked potentials. The effects of the stimulus order and inter-train interval (ITI) on the amplitudes are shown. The amplitudes (mean±standard deviation) are averaged and normalized to the first ones in the train (dashed line). Asterisks mark the amplitudes that differ from the first one (*p*<0.05) in the train according to the pairwise comparisons using Bonferroni adjustment of *p*-values for correction of multiple comparisons. A) All 4-pulse sequences. B) ITI 1 s. C) ITI 3 s. D) ITI 4 s. E) ITI 6 s. F) ITI 7 s. G) ITI 12 s. H) ITI 17 s. I) 20-pulse sequence. J) 120 pulses/train at inter-stimulus interval (ISI) of 1 s.

The RS effect was observed as an average decrease of 20–50% in the normalized MEP amplitudes (*p*<0.001, Fig 2). The stimulus number (*p*<0.001, *F* = 78.73) and ITI (*p*<0.001, *F* = 23.79) affected the amplitudes in the 4-pulses/train sequences. RS continued even to the 20th MEP (*p*<0.001) in the long trains. The amplitude seemed to recover gradually after the second MEP but did not reach the level of the first MEP during the trains.

The effect of the ITI on the MEP amplitudes is shown in Fig 2. With long ITIs (7–17 s), the RS appeared to be more pronounced; at ITI of 4 s, the normalized MEP amplitudes were higher than at ITIs of 7 s (*p*<0.001), 12 s (*p* = 0.003), and 17 s (*p* = 0.001). On average, the normalized MEP amplitudes in long-ITI sequences were 52±28% (2nd, mean±standard deviation), 59±28% (3rd), and 63±30% (4th) of the normalized first MEP amplitude, whereas with shorter ITIs (3–6 s), the normalized amplitudes were 70±28% (2nd), 69±26% (3rd), and 74±26% (4th) of the first amplitude. The variability of normalized MEPs varied slightly with different ITIs being lowest with ITI of 1 s and highest with ITI of 12 s.

ITI had an effect on the amplitudes of the first MEPs in the trains (*p*<0.001, *F* = 15.89, Table 1). On average, the first MEPs were smaller in amplitude with an ITI of 3 s than at the other ITIs, the difference being significant with ITIs of 4, 6, 7, and 17 s (*p*≤0.001). In addition, the first MEPs were smaller with an ITI of 12 s when compared to an ITI of 17 s (*p* = 0.015). With an ITI of 4 s, the first MEPs were generally greater than those with ITIs of 3, 6, and 12 s (*p*<0.05). Further, with an ITI of 4 s, all MEPs in the long train of 20 pulses appeared to be greater than those with ITIs of 3 s, 6 s, and 12 s (*p*<0.05). The first MEP amplitudes of the trains remained unchanged within the sequences (*p* = 0.880).

**Table 1:** Differences between the first motor-evoked potential amplitudes with different inter-train intervals.

	ITI 1 s	ITI 3 s	ITI 4 s	ITI 6 s	ITI 7 s	ITI 12 s	ITI 17 s
**ITI 1 s**	**–**	25 *p* = 1.000	**-641 *p*<0.001**	**-349 *p* = 0.005**	**-484 *p*<0.001**	-247 *p* = 0.222	**-566 *p*<0.001**
**ITI 3 s**	-25 *p* = 1.000	**–**	**-666 *p*<0.001**	**-374** ***p* = 0.001**	**-509** ***p*<0.001**	-272 *p* = 0.082	**-591** ***p*<0.001**
**ITI 4 s**	**641 *p*<0.001**	**666 *p*<0.001**	**–**	**292** ***p* = 0.033**	158 *p* = 1.000	**395** ***p* = 0.001**	76 *p* = 1.000
**ITI 6 s**	**349 *p* = 0.005**	**374 *p* = 0.001**	**-292 *p* = 0.033**	**–**	-135 *p* = 1.000	102 *p* = 1.000	-217 *p* = 0.390
**ITI 7 s**	**484 *p*<0.001**	**509 *p*<0.001**	-158 *p* = 1.000	135 *p* = 1.000	**–**	237 *p* = 0.247	-82 *p* = 1.000
**ITI 12 s**	247 *p* = 0.222	272 *p* = 0.082	**-395 *p* = 0.001**	-102 *p* = 1.000	-237 *p* = 0.247	**–**	**-319** ***p* = 0.015**
**ITI 17 s**	**566 *p*<0.001**	**591 *p*<0.001**	-76 *p* = 1.000	217 *p* = 0.390	82 *p* = 1.000	**319 *p* = 0.015**	**–**

Mean differences between the first motor-evoked potential (MEP) amplitudes (μV) in the trains with different inter-train intervals (ITI). The differences were obtained by subtracting the MEP amplitudes with ITIs shown in the first row from the MEP amplitudes with ITIs shown in the first column. Bonferroni corrected pairwise comparison *p*-values for the differences are also shown. *p*<0.05 are shown in bold.

RS was not observed with the ITI of 1 s, i.e., when ITI was equal to ISI (Fig 2B). Hence, the first MEP amplitude did not differ from the 2nd–4th MEP amplitudes (*p* = 1.000). The mean normalized MEP amplitudes of the 120-pulse long sequence as a function of the stimulus number are shown in Fig 2J.

## Discussion

We studied the effect of ITI on the appearance and effect size of RS. Agreeing with our hypothesis, RS was found in MEPs with all ITIs except an ITI of 1 s, indicating that ITI needs to be longer than the ISI. However, according to these results, ITI does not necessarily need to be as long as the previously used 17 s [7,11,12] which would be advantageous as short ITIs would speed up the examination protocol. However, with long ITIs, RS seemed to be more pronounced suggesting that it would be beneficial to use an ITI of at least 6 s in order to ensure a stable and clear assessment of the RS effect.

The first MEPs in the trains remained unchanged within the sequences. Nevertheless, the first MEP amplitudes were different with different ITIs. The ITIs of 3 and 4 s had dissimilar effects on the first MEP amplitudes. With an ITI of 3 s, the first MEPs were smaller in amplitude in comparison with other ITIs, whereas with an ITI of 4 s, they were larger. Therefore, the MEPs with short ITIs appear to be less reliable for measuring RS compared with the longer ITIs as carry-over effects may appear with short ITIs. This implies that future RS studies should be performed with ITIs of at least 6 s to avoid potential carry-over effects. Carry-over effects may affect other common TMS measures as well, and hence sufficient trial-to-trial interval should be applied [5]. In a previous study, the first silent periods in the trains were observed to be longest at the beginning of RS stimulation sequences and therefore RS protocol might induce longer-term effects which are not, however, seen in MEPs of a resting muscle [12].

The origin of RS is still unknown and several mechanisms have been considered in past studies [16,17]. For example, a sharpening model, whereby fewer neurons react to the stimuli, might have a role in RS [7,25]. Another possible explanation is that RS is evoked by an inhibitory feedback from the somatosensory cortex [7]. In addition, RS might involve a similar GABA-related mechanism as has been claimed to mediate short-interval intra-cortical inhibition and long-interval intra-cortical inhibition induced with paired-pulse TMS [12,26–28]. Moreover, GABA and RS might be connected as cortical GABA release and motor activity were negatively correlated in a study examining habituation in rats [29]. RS is likely a general short-term mechanism and a normal reaction to repeated stimuli, whereas longer-lasting effects of low-frequency repetitive TMS are related to long-term depression. With short ITIs, however, the protocol resembles low-frequency repetitive TMS, and therefore, long-term depression might influence RS. Previously, ITI has been observed to have an effect on the plastic effects of high-frequency repetitive TMS [6]. The mechanisms of this effect are most likely different from RS mechanisms and RS is not associated to plasticity. In other forms of habituation, glutamate and dopamine neurotransmissions might be involved [30]. Furthermore, the high-amplitudes of the first MEPs in the trains may also be explained by a startle reaction. The potential startle also raises another question, whether it is something that is generally measured through common single-pulse MEPs, and whether it is one of the fundamental reasons, why MEP amplitudes exhibit enormous variation from trial to trial and from individual to individual, and why potentially the first response in the train of MEPs should be excluded from analyses. However, most likely RS is mediated through several different cellular mechanisms in different areas of the nervous system [15]. The mechanisms may be dependent on the protocol parameters such as stimulus types and times.

RS was found to continue even after 20 pulses, which is in agreement with the findings where RS has been measured from AEPs [8]. Nevertheless, the MEPs began to increase in amplitude after the second stimulus demonstrating partial recovery. A total recovery of MEPs to the level of the first MEP in the train was not observed indicating a slow recovery rate. When the mean amplitudes were extrapolated with a first-degree polynomial, the MEPs would have reached the level of the first MEP after 45 pulses.

In the continuous 120-pulse train, the single-trial MEPs quickly reached the level of the first MEP when averaged over all subjects; in the grand average curve, the sixth MEP amplitude was already at the level of the first one. The inter-subject variability in these 120-pulse trains was high; in three subjects, RS lasted throughout the entire sequence (i.e., after the first MEP, all MEP amplitudes in the train were lower than the first MEP), in five subjects the MEPs recovered rapidly and exceeded the first MEP amplitude, and in the remaining subjects, the amplitudes varied (Fig 2J). RS is not seen in this sequence likely due to the high inter-subject variability and due to the fact, that only single-trial MEPs were compared in the individual level, and the amplitudes shown represent single-trial MEP amplitudes. The RS effect may be covered by the characteristically high variation in MEP amplitudes, as no repeated trials were analyzed to reduce the variation. Moreover, this sequence was applied at the end of the experiment and might be influenced by reduced excitability of the subjects. It has been reported that 1 Hz repetitive TMS often has an inhibitory effect on the MEP amplitudes [4,24], but inconsistent reports exist [31]. However, in addition to the frequency, also the duration of the train and SI influence the effect of repeated TMS [4].

One limitation of the present study is that the full recovery of MEPs could not be demonstrated without at least 3 s break between trains. Therefore, pulse trains longer than 45 pulses should likely be used if one wishes to evaluate the full recovery of the MEP amplitudes in RS. In addition, either four or twenty pulses in a train were given and only an ISI of 1 s was applied; different train durations and ISIs should be investigated in the future. This compromise was made to avoid excessively long study sessions. We used four pulses in the trains in order to evoke a clear and reliable effect that would last beyond two stimuli. Four pulses have also been used in previous studies to demonstrate a prolonged RS effect [7,11,12,21]. Interaction effects between ITI and train duration might exist; in the future, these could be studied by investigating the duration of the train with different ITIs. Potentially in this way, the hyperexcitability component caused by the arousal effect on the first pulse could be better identified and compared with the inhibitory component. Another limitation is that the effect of SI was not investigated. It is commonly assumed that the RS is faster and more prominent with lower than higher intensities of stimulation [15] and a similar effect might also be found in RS of MEPs. We considered these limitations as acceptable to keep the duration of the experiments reasonable. Furthermore, the induced electric field direction was optimized individually for each subject to maximize the MEPs. This may have influenced the results by optimizing and selecting the neuronal population which was stimulated [32,33]. However, based on the maximal MEPs induced with these directions, it seems likely that the activated neurons were directly involved in the execution of movements.

In conclusion, ITI was found to have an effect on the appearance of RS; RS was observed at ITIs of 3–17 s, but not with an ITI of 1 s. We found that the RS effect continued to suppress MEP amplitudes up to the maximum number of tested 20 stimuli and a full recovery of RS could not be demonstrated. In the future, ITIs shorter than 17 s may be used to optimize study routines for motor system RS with TMS. Very short ITIs should be avoided, as they may weaken the observed RS. The findings of the present study will be beneficial in the planning of future study protocols to enable a quick and reliable assessment of the motor cortical RS, and provide additional information on RS phenomenon in the healthy brain.

## Supporting information

S1 Appendix Data. Motor-evoked potential (MEP) amplitudes induced with transcranial magnetic stimulation in trains of four or twenty pulses with different inter-train intervals (ITIs).
(XLSX)
